# Two-dimensional *versus* three-dimensional laparoscopy in surgical efficacy: a systematic review and meta-analysis

**DOI:** 10.18632/oncotarget.10916

**Published:** 2016-07-29

**Authors:** Ji Cheng, Jinbo Gao, Xiaoming Shuai, Guobin Wang, Kaixiong Tao

**Affiliations:** ^1^ Department of Gastrointestinal Surgery, Union Hospital, Tongji Medical College, Huazhong University of Science and Technology, Wuhan, Hubei, China

**Keywords:** three-dimensional laparoscopy, two-dimensional laparoscopy, surgical efficacy, systematic review, meta-analysis

## Abstract

**Background:**

Laparoscopy is a revolutionary technique in modern surgery. However, the comparative efficacy between two-dimensional laparoscopy and three-dimensional laparoscopy remains in uncertainty. Therefore we performed this systematic review and meta-analysis in order to seek for answers.

**Methods:**

Databases of PubMed, Web of Science, EMBASE and Cochrane Library were carefully screened. Clinical trials comparing two-dimensional versus three-dimensional laparoscopy were included for pooled analysis. Observational and randomized trials were methodologically appraised by Newcastle-Ottawa Scale and Revised Jadad's Scale respectively. Subgroup analyses were additionally conducted to clarify the potential confounding elements. Outcome stability was examined by sensitivity analysis, and publication bias was analyzed by Begg's test and Egger's test.

**Results:**

21 trials were screened out from the preliminary 3126 records. All included studies were high-quality in methodology, except for Bilgen 2013 and Ruan 2015. Three-dimensional laparoscopy was superior to two-dimensional laparoscopy in terms of surgical time (*P* < 0.00001), blood loss (*P* = 0.01), perioperative complications (*P* = 0.04) and hospital stay (*P* = 0.03). Additionally, both techniques demonstrated comparable results of secondary endpoints, including drainage volume (*P* = 0.74), drainage time (*P* = 0.26), numbers of retrieved lymphnodes (*P* = 0.85), hospital expenses (*P* = 0.49), anastomosis time in prostatectomy (P=0.15) and 6-month continence rate (*P* = 0.61). The pooled outcomes of primary endopoints were verified to be stable by sensitivity analysis. Although Begg's test (*P* = 0.215) and Egger's test (*P* = 0.003) revealed that there was publication bias across included studies, Trim-and-Fill method confirmed that the results remained stable.

**Conclusion:**

Three-dimensional laparoscopy is a preferably surgical option against two-dimensional laparoscopy due to its better surgical efficacy.

## INTRODUCTION

Since its clinical debut in 1987 for a patient undergoing cholecystectomy, laparoscopic arm has emerged as a catalyst of surgical renovation during the past three decades, which rapidly spreads its application to the entire abdominal operations [1]. Traditional two-dimensional (2D) laparoscopy features higher definition of graphic display and more visional comforts, as well as lower threshold expenditure. Nevertheless, lacking of stereoscopic perception not only leads to elongated learning curves among surgical novices, but also endangers the estimate of surgical depth during critical operations, especially the current trend for laparoscopy is moving towards deeper and riskier surgical regions such as radical pancreatectomy and prostatectomy [2]. Therefore, a three-dimensional (3D) view with better stereoscopic demonstration is urgently needed.

In 1993, Wenzl et al [3] firstly implemented a gynecological operation under a laparoscopic 3D instrument. However, the initial 3D display was mainly based on Shutter Glass (SG) technique, which provided poor-definition images and was harmful to surgeons' eyes. Owing to the manufactural improvements in optic industry, 3D laparoscope characterized by Film-type Patterned Retarder (FPR) was subsequently invented. This new generation of 3D laparoscopic facility features high-definition and stable image, alleviating the visional burdens of surgical operators and truly bringing laparoscopic operations into a tridimensional era. Therefore, Buchs et al [4] firstly reported a smooth operation by FPR glasses in 2012, and from then on, 3D laparoscopy began globally popularized among surgeon communities including China [5].

Unfortunately, the comparative efficacy of 3D laparoscopy against 2D laparoscopy remains undetermined, due to the scarcity of clinical evidences especially a systematic summary of surgical indicators. Hence based on current literatures, we performed this systematic review and meta-analysis in order to explore the comparative efficacy of 3D laparoscopy in abdominal operations.

## RESULTS

### General characteristics

**Figure 1:**
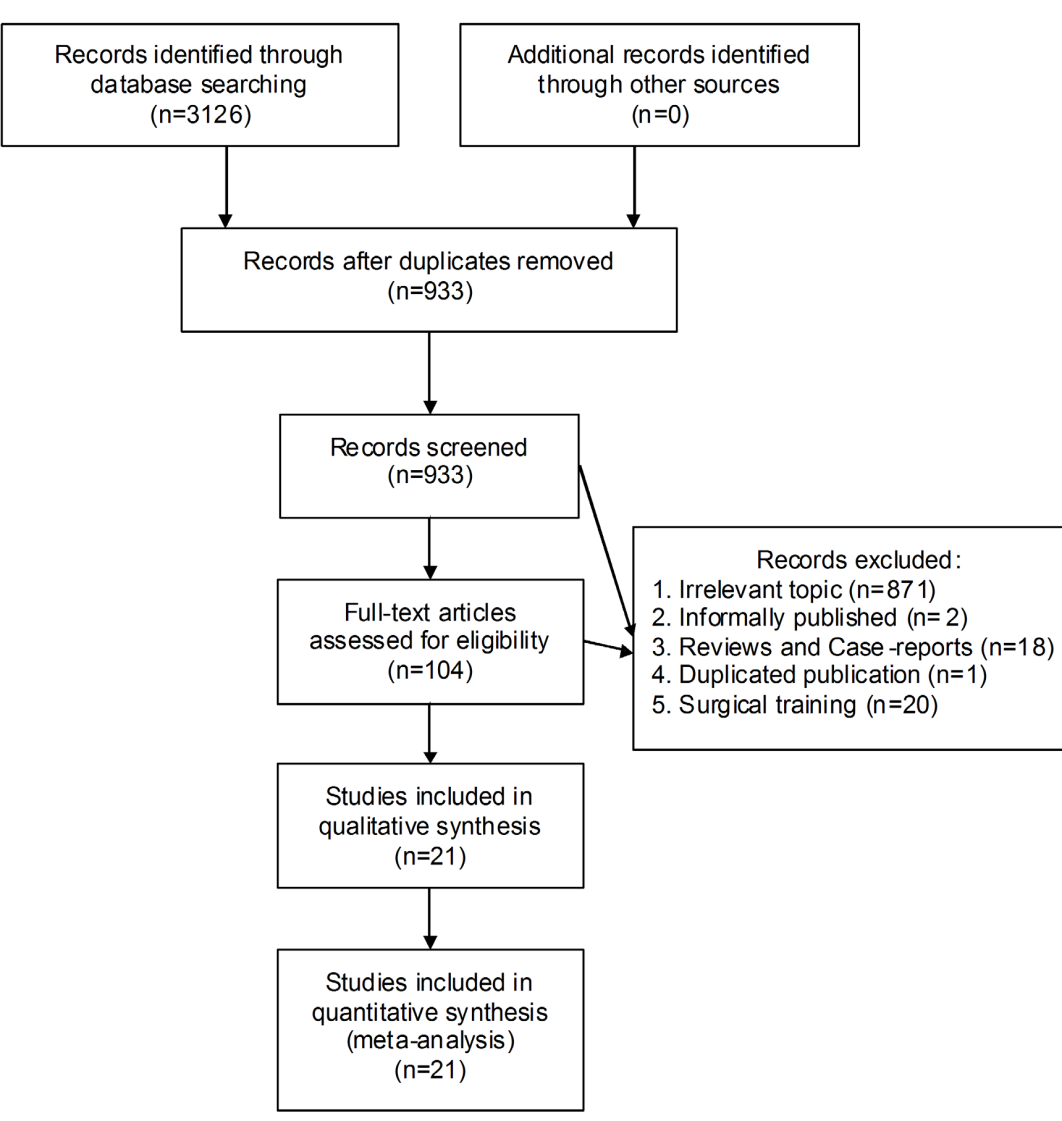
Selection flow chart of our meta-analysis

Among 3126 retrieved records, 21 studies were included into the quantitative analysis (Figure 1). 12 investigations were written in English while the remaining was published in Chinese (*n* = 9). China was the chief source region of eligible trials (*n* = 10), followed by Italy (*n* = 5) and Turkey (*n* = 3). A total of 13 studies were retrospectively conducted, while 8 trials were randomly designed. The most frequent surgical type was cholecystectomy (*n* = 4) and prostatectomy (*n* = 4). The total amount of sample-size was 1520 (two-dimensional: 819; three-dimensional: 701), individually ranging from 22 to 154. According to the statistical analysis of demographic parameters (age, sex ratio), included studies were confirmed to be internally comparable (*P* > 0.05) (Table 1).

**Table 1:** Demographic characteristics of included studies

Study	Country	Trial type	Surgical type	Group	Sample-size	Age (Y)	Sex (M/F)
Agrusa 2015 [6]	Italy	Retrospective	Adrenalectomy	2D	26	54.3±9.0	17/9
				3D	13	55.8±7.5	8/5
Aykan 2014 [7]	Turkey	Retrospective	Prostatectomy	2D	66	64.5±8.0	66/0
				3D	29	65.0±6.0	29/0
Bilgen 2013 [8]	Turkey	Randomized	Cholecystectomy	2D	11	53.0	1/10
				3D	11	54.0	0/11
Bove 2015 [9]	Italy	Retrospective	Prostatectomy	2D	43	60.1	43/0
				3D	43	63.9	43/0
Chen 2014 [10]	China	Retrospective	Gastrectomy	2D	40	51.0±5.2	30/10
				3D	40	49.0±4.8	27/13
Chen 2015 [11]	China	Retrospective	Thyroidectomy	2D	34	49.2±11.6	6/28
				3D	26	46.2±11.7	6/20
Chen 2016 [12]	China	Randomized	Ureterotomy	2D	20	45.8±12.3	10/10
				3D	25	41.6±13.2	14/11
Curro 2015-1 [13]	Italy	Randomized	Gastric bypass	2D	20	38.0±8.8	4/16
				3D	20	39.0±9.5	4/16
Curro 2015-2 [13]	Italy	Randomized	Sleeve gastrectomy	2D	20	36.0±8.3	3/17
				3D	20	36.0±9.8	4/16
Curro 2016 [14]	Italy	Retrospective	Colectomy	2D	25	68.0±8.0	14/11
				3D	25	69.0±9.5	12/13
Hanna 1998 [15]	UK	Randomized	Cholecystectomy	2D	30	52.0±15.0	8/22
				3D	30	58.0±11.8	7/23
Hou 2015 [16]	China	Randomized	Esophagectomy	2D	76	55.1±7.6	44/32
				3D	78	55.7±6.3	41/37
Ji 2014 [17]	China	Retrospective	Rectectomy	2D	20	59.0±8.0	15/5
				3D	16	55.0±8.0	9/7
Kinoshita 2015 [18]	Japan	Randomized	Prostatectomy	2D	57	65.9±4.7	57/0
				3D	59	66.5±4.5	59/0
Navarra 2015 [19]	Italy	Randomized	Cholecystectomy	2D	35	50.0±10.5	9/26
				3D	35	56.0±9.8	7/28
Ruan 2015 [20]	China	Randomized	Nephrectomy	2D	45	58.7±3.2	22/23
				3D	45	60.4±2.7	24/21
Usta 2014 [21]	Turkey	Retrospective	Hysterectomy	2D	91	52.2	0/91
				3D	56	49.5	0/56
Velayutham 2016 [22]	France	Retrospective	Hepatectomy	2D	40	NA	24/16
				3D	20	NA	9/11
Xu 2014 [23]	China	Retrospective	Pyeloplasty	2D	15	31.0±6.0	7/8
				3D	16	30.0±6.0	9/7
Xu 2015 [24]	China	Retrospective	Prostatectomy	2D	32	67.8±8.4	32/0
				3D	18	67.3±6.6	18/0
Zeng 2016 [25]	China	Retrospective	Cholecystectomy	2D	43	57.0±12.0	28/15
				3D	46	59.0±11.0	28/18
Zou 2014 [26]	China	Retrospective	Thyroidectomy	2D	30	44.4±7.6	12/18
				3D	30	43.3±7.8	10/20

Y: years; M/F: male/female; NA: not available; 2D: two-dimensional; 3D: three-dimensional;

### Methodological quality

By Newcastle-Ottawa Scale, all retrospective studies were confirmed as high-quality trials in methodology (NOS>6) (Table 2). Moreover, by Revised Jadad's Scale, the majority of randomized trials were methodologically rigorous, except for Bilgen 2013 and Ruan 2015 (lower than 4 points) (Table 3).

**Table 2:** Methodological assessment by Newcastle-Ottawa Scale

Study	Selection	Comparability	Outcome	Total
Agrusa 2015	3	2	2	7
Aykan 2014	3	2	2	7
Bove 2015	3	2	3	8
Chen 2014	3	2	1	6
Chen 2015	3	2	1	6
Curro 2016	3	2	1	6
Ji 2014	3	2	2	7
Usta 2014	3	2	2	7
Velayutham 2016	3	2	2	7
Xu 2014	3	2	3	8
Xu 2015	3	2	1	6
Zeng 2016	3	2	1	6
Zou 2014	3	2	1	6

**Table 3:** Methodological assessment by Revised Jadad's Scale

Study	Randomization	Allocation concealment	Blindness	Withdrawal	Total
Bilgen 2013	2	1	0	0	3
Chen 2016	2	1	0	1	4
Curro 2015	2	2	0	1	5
Hanna 1998	2	2	0	1	5
Hou 2015	2	1	0	1	4
Kinoshita 2015	2	2	0	1	5
Navarra 2015	2	2	0	0	4
Ruan 2015	1	1	0	1	3

### Primary endpoint-surgical time

#### Overall

**Figure 2:**
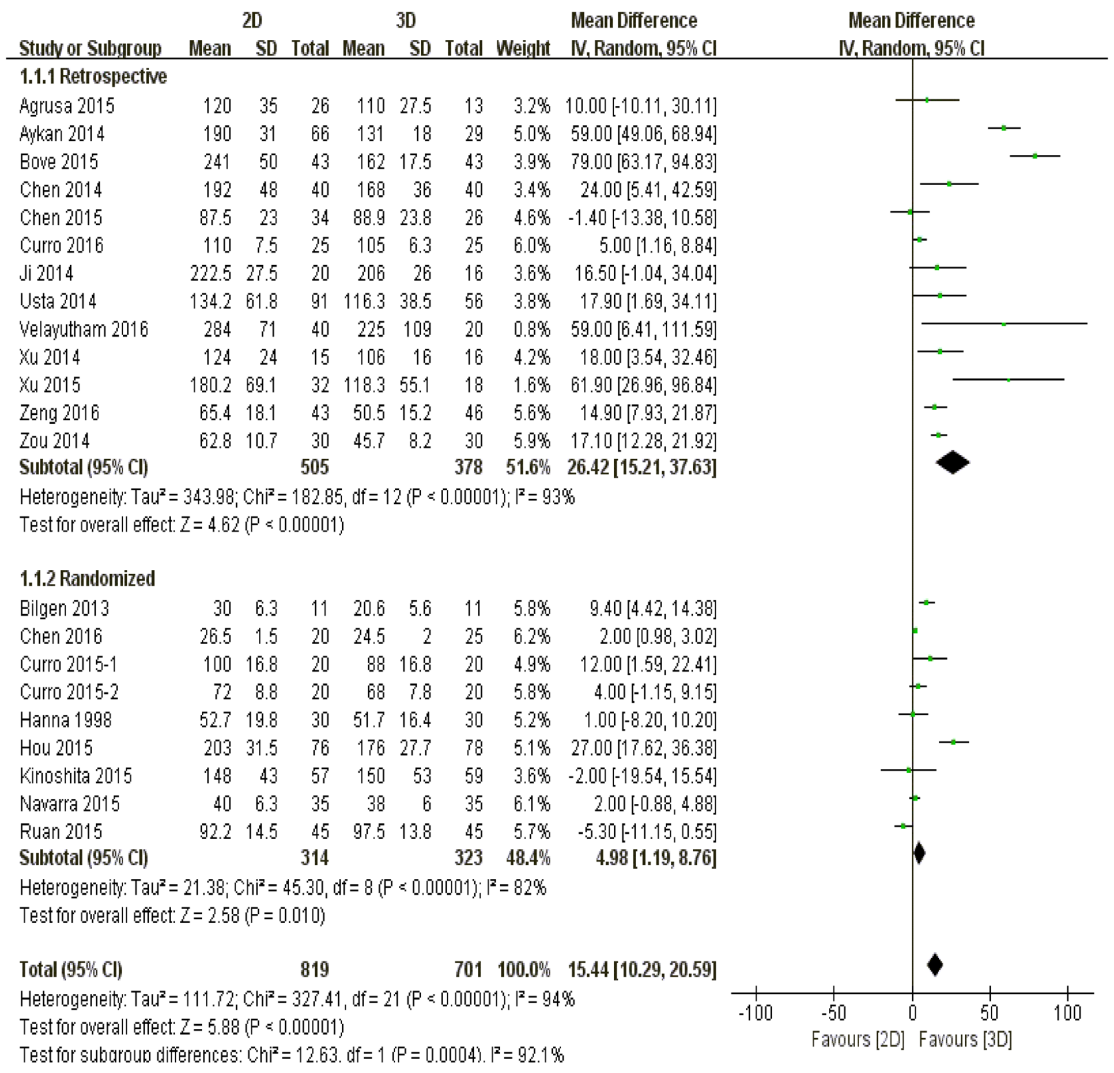
The comparison of surgical time according to different study types

The surgical duration by 3D laparoscopy was much lower than that of 2D technique (*P* < 0.00001) (Figure 2).

#### Study type

No matter the studies were retrospectively (*P* < 0.00001) or randomly designed (*P* < 0.01), three-dimensional laparoscopy spent significantly less surgical time than two-dimensional did (Figure 2).

#### Surgical type

**Figure 3:**
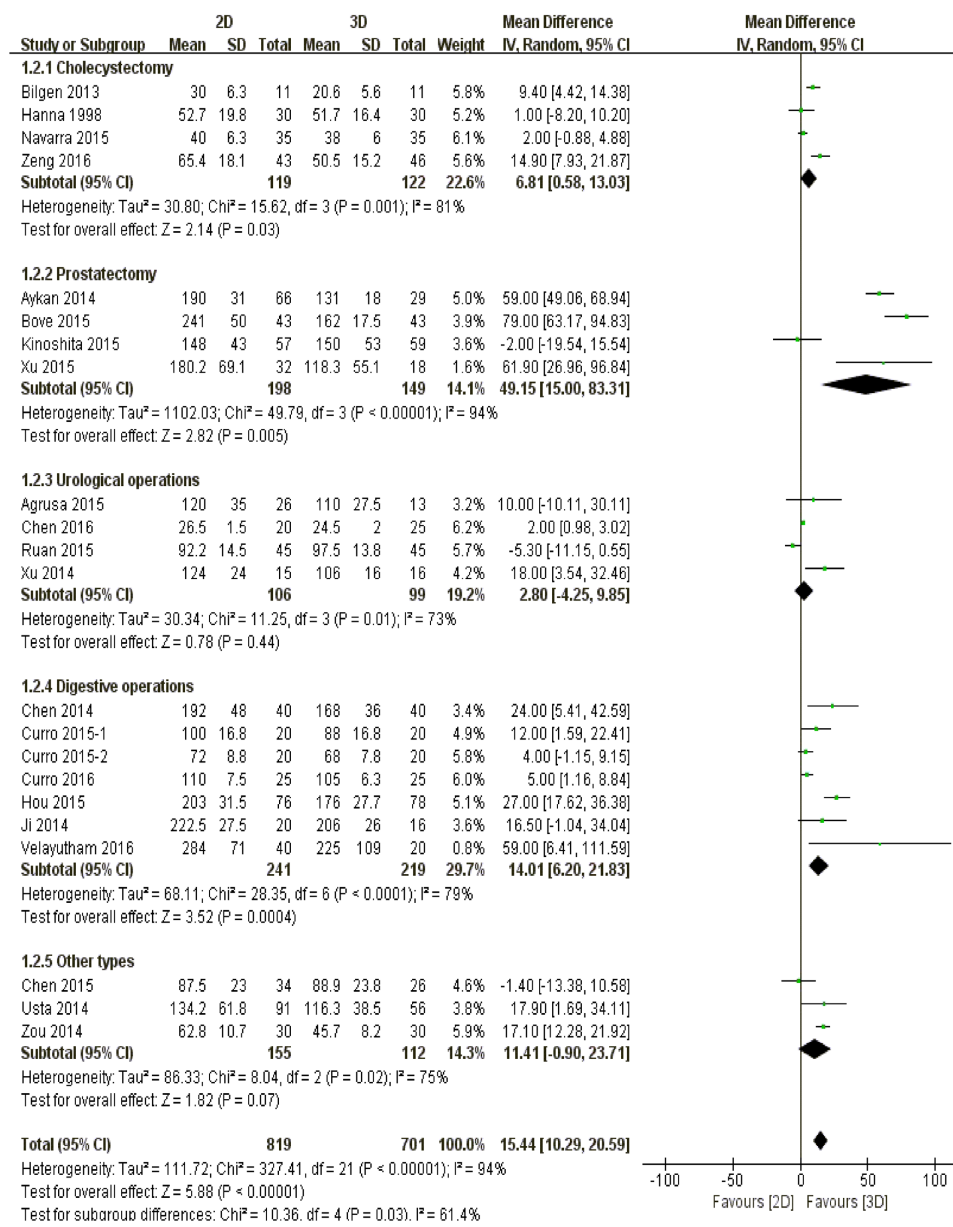
The comparison of surgical time according to different surgical types

Patients undergoing cholecystectomy (*P* = 0.03), prostatectomy (*P* = 0.005) and digestive operations (*P* = 0.0004) endured less surgical time by three-dimensional laparoscopy than those by two-dimensional device. On the other hand, there was no significant difference between 3D and 2D laparoscopy in terms of urological operations (*P* = 0.44) and other types (*P* = 0.07) (Figure 3).

### Primary endpoint-blood loss

#### Overall

**Figure 4:**
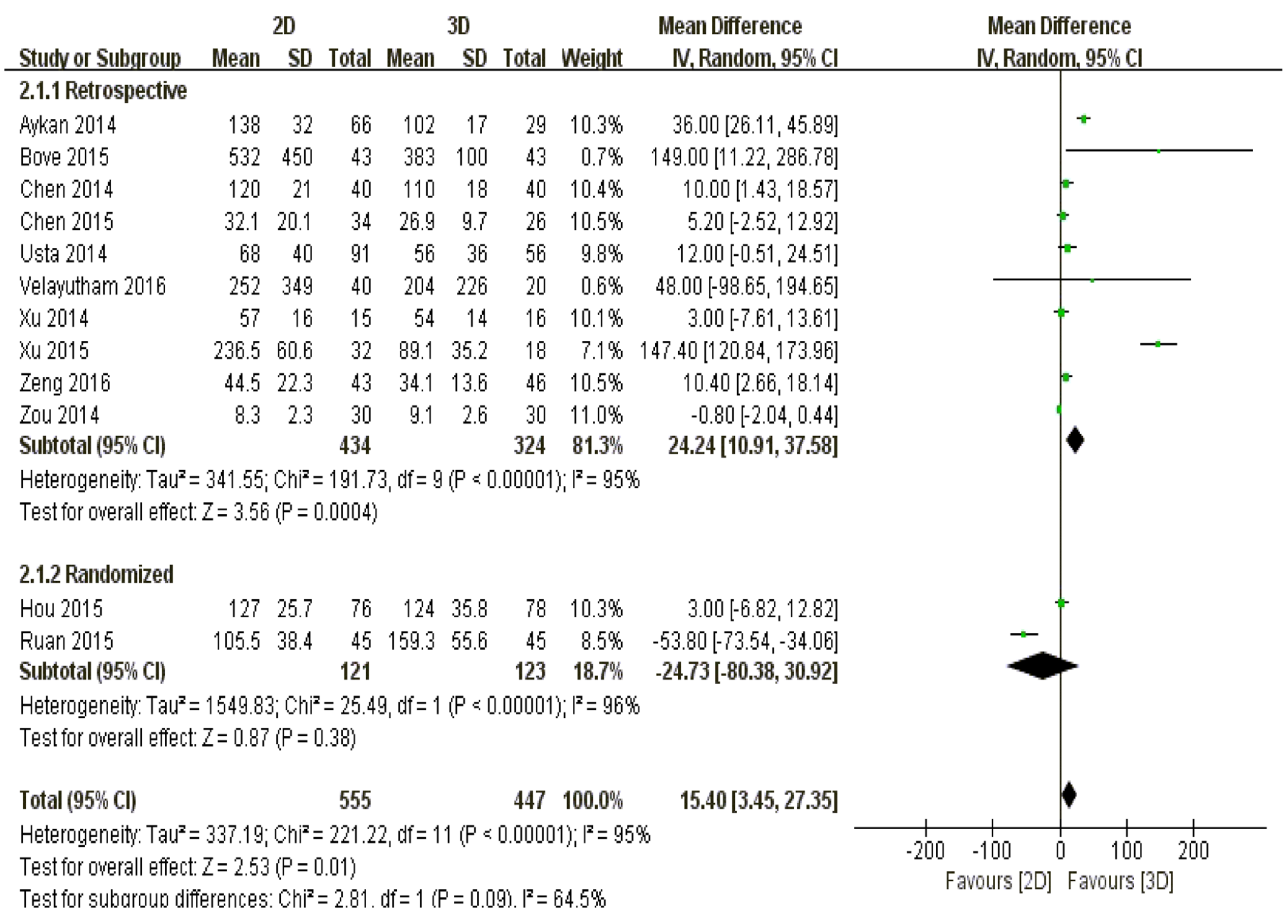
The comparison of blood loss according to different study types

Lower volume of intraoperative blood loss was observed among 3D group than that of 2D group (*P* = 0.01) (Figure 4).

#### Study type

It was retrospectively confirmed that 3D laparoscopy led to less blood loss against 2D laparoscopy (*P* = 0.0004), while randomized investigations summarized that patients undergoing both techniques had comparable volume of blood loss during operations (*P* = 0.38) (Figure 4).

#### Surgical type

**Figure 5:**
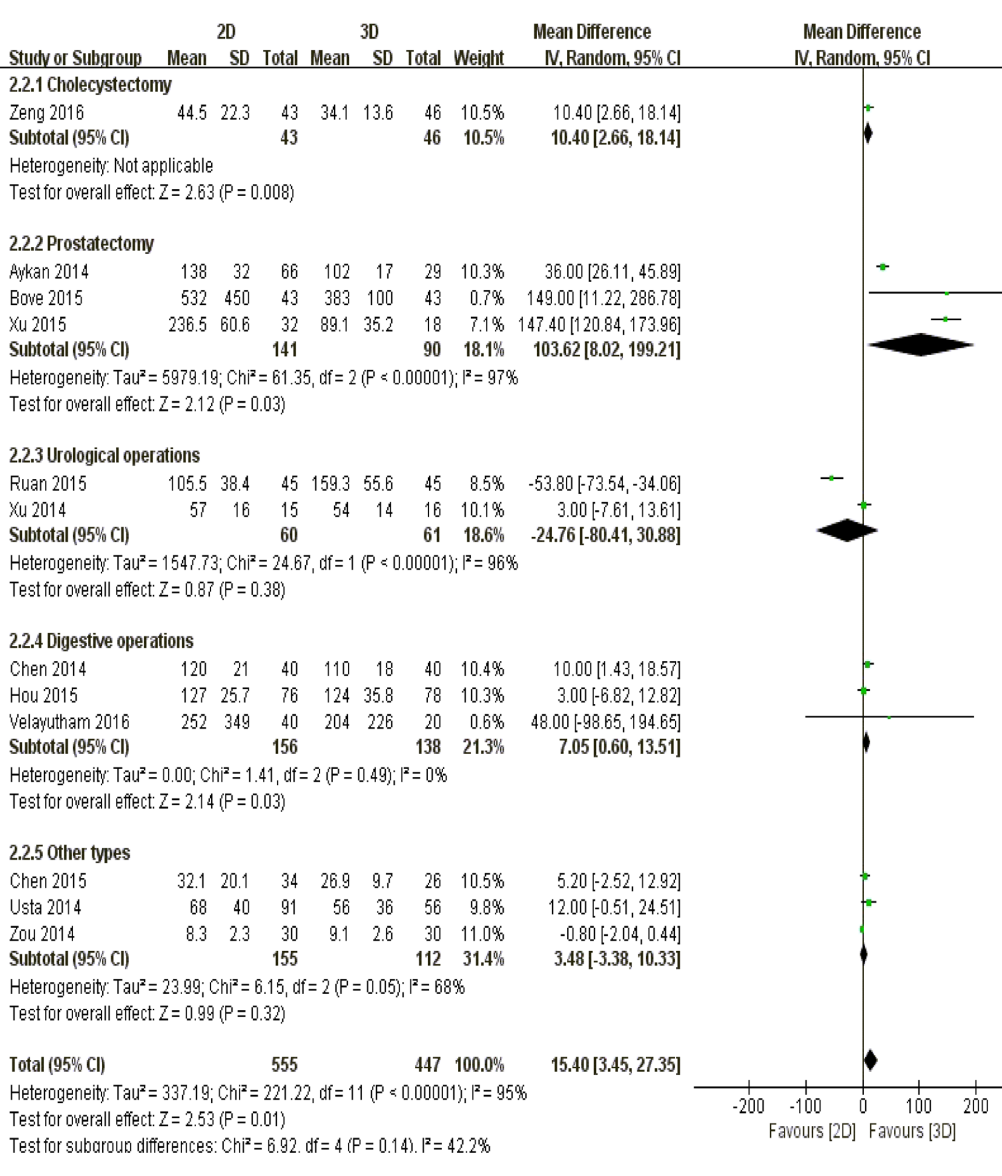
The comparison of blood loss according to different surgical types

Patients undergoing cholecystectomy (*P* = 0.008), prostatectomy (*P* = 0.03) and digestive operations (P = 0.03) suffered less intraoperative blood loss by 3D laparoscopic arm. However, both techniques resulted in similar magnitude of blood loss amid patients with urological (*P* = 0.38) or other types of diseases (*P* = 0.32) (Figure 5).

### Primary endpoint-perioperative complications

#### Overall

Patients had lower incidence of perioperative complications following 3D management than those of 2D group (*P* = 0.04) (Supplementary Figure S1).

#### Study type

In terms of perioperative complications, there was no significant difference between 3D and 2D laparoscopy, regardless of retrospective (*P* = 0.07) or randomized studies (*P* = 0.29) (Supplementary Figure S1).

#### Surgical type

Comparable incidence of perioperative complications was observed between 3D and 2D laparoscopy, whichever of cholecystectomy (*P* = 0.93), prostatectomy (*P* = 0.05), urological operations (*P* = 0.18), digestive operations (*P* = 0.87) or other types (*P* = 0.37) (Supplementary Figure S2).

### Primary endpoint-hospital stay

#### Overall

Patients receiving 3D laparoscopic management experienced shorter period of hospital stay than those with two-dimensional intervention (*P* = 0.03) (Supplementary Figure S3).

#### Study type

The pooled analysis of retrospective studies suggested that 3D laparoscopy was more effective in reducing hospital stay than 2D laparoscopy was (*P* = 0.04). However, evidence from randomized trials revealed a similar hospital stay among patients undergoing both interventions (*P* = 0.58) (Supplementary Figure S3).

#### Surgical type

Patients between 3D and 2D group had similar length of hospital stay, including those undergoing cholecystectomy (*P* = 0.58), urological operations (*P* = 0.39), digestive operations (*P* = 0.90) and other types of operations (*P* = 0.05). Nevertheless, 3D laparoscopic prostatectomy resulted in shorter hospital stay against 2D technique (*P* = 0.04) (Supplementary Figure S4).

### Primary endpoint-conversion rate

#### Overall

The conversion rate between three-dimensional and two-dimensional laparoscopy was statistically equivalent (*P* = 0.68) (Supplementary Figure S5).

#### Study type

The pooled outcome of retrospective trials revealed that both 3D and 2D interventions resulted in similar conversion rate (*P* = 0.68). Data of randomized studies was not estimable (Supplementary Figure S5).

#### Surgical type

Both 3D and 2D techniques led to similar conversion rate among patients undergoing cholecystectomy (*P* = 0.63), digestive operations (*P* = 0.62) and other types of operations (*P* = 0.60). Data of prostatectomy and urological operations was not estimable (Supplementary Figure S6).

### Secondary endpoints

#### Drainage volume

The drainage volume of surgical patients was quantitatively identical between three-dimensional and two-dimensional group (*P* = 0.74) (Supplementary Figure S7).

#### Drainage time

There was no significant difference of drainage time between 3D and 2D techniques (*P* = 0.26) (Supplementary Figure S8).

#### Numbers of retrieved lymph nodes

Comparable amount of lymph nodes was retrieved irrespective of tridimensional and two-dimensional laparoscopy (*P* = 0.85) (Supplementary Figure S9).

#### Hospital expenses

The average hospital expenses between 2D and 3D laparoscopy were statistically equivalent (*P* = 0.49) (Supplementary Figure S10).

#### Anastomosis time in prostatectomy

The pooled result suggested that there was no significant difference of anastomosis time between 2D and 3D interventions (P = 0.15) (Supplementary Figure S11).

#### 6-month continence rate

Patients had similar 6-month continence rate regardless of 2D or 3D laparoscopy (*P* = 0.61) (Supplementary Figure S12).

### Sensitivity analysis

Firstly, the sensitivity analysis was performed by excluding low-quality trials. Despite Bilgen 2013 and Ruan 2015 were eliminated from primary endpoint analysis, the majority of pooled outcomes remained stable, except for perioperative complications (P value changing from 0.04 to 0.06).

Secondly, by interchanging fixed-effects and random-effects models, the pooled results of primary endpoints were confirmed to be stable, except for blood loss (P value changing from 0.01 to 0.29)

Thirdly, by randomly excluding one trial from pooled analysis in STATA 12.0, the outcome of surgical time was verified to be stable (Supplementary Figure S13).

### Publication bias

We took surgical time as an exemplary indicator for publication bias assessment. P values of Begg's test (Supplementary Figure S14) and Egger's test (Supplementary Figure S15) were 0.215 and 0.003 respectively, revealing a potential existence of publication bias across included studies. Thus we additionally carried out a Trim-and-Fill method, whose result indicated that no studies were trimmed or filled and the outcome was therefore stable (Supplementary Figure S16).

## DISCUSSION

According to the pooled outcomes of primary endpoints, three-dimensional laparoscopy resulted in significantly less surgical time, blood loss, perioperative complications and hospital stay among surgical patients. It is relatively comprehensible that its overwhelming preponderance against 2D laparoscopy may mainly attribute to the more stereoscopic surgical view. A tridimensional reconstruction of target region greatly facilitates the estimate of anatomic depth and accuracy of surgical manipulation [27]. Meanwhile, despite of wearing 3D glasses, current technological improvements successfully prevent surgical operators from visual fatigue. However, in terms of conversion rate, 3D laparoscopy resulted in similar outcome compared to 2D technique. This is probably because that although two-dimensional image leads to higher risk of surgical errors, those potential mistakes are still unable to threaten the overall operative safety in most circumstances, especially among surgeons with rich experiences and advanced skills [28–30]. Therefore a traditional laparoscopy is quite enough to deal with the intraoperative accidents. Furthermore, based on different study types and surgical types, subgroup analysis had brought more specific evidences besides the overall comparisons. Due to its significant advantages on primary endpoints, 3D laparoscopy was strongly recommended for cholecystectomy and prostatectomy. It is known to all that a neat dissection of Calot's triangle and a functional reservation of surrounding structures are crucial procedures for a successful cholecystectomy and prostatectomy respectively. A more stereoscopic visual perception greatly supports tissue separation and vessel ligation. On the other hand, regardless of 2D or 3D laparoscopy, comparable efficacy was observed among patients undergoing digestive operations or urological operations, which was theoretically abnormal and hence more convincing literatures were still needed for future supplements.

Including drainage volume, drainage time, numbers of retrieved lymph nodes, hospital expenses, anastomosis time in prostatectomy and 6-month continence rate, both techniques displayed statistical similarity on secondary endpoints. Generally, the advantage of security could directly result in better outcomes of these secondary parameters. We assume that these exceptional consequences may blame on the limited amount of included studies, which diminishes the outcome credibility.

Currently, conclusive evidences that analyze the comparative efficacy of tridimensional laparoscopy remain in scarcity. Sorensen et al [31] performed a systematic review of 3D laparoscopy versus 2D laparoscopy on simulated settings. Without examining the clinical significance, their results merely revealed a better performance on surgical tasks and trainings by tridimensional laparoscopy. Sakata et al [2] systematically summarized the technical advantages of current 3D laparoscopy, implicating the great potential of its surgical application. By far, large-scale randomized trial of this topic is still lacking and no consensus has been reached among current literatures. Hou et al [16], a randomized study of 154 participants, concluded that both techniques achieved similar outcomes of primary endpoints. However, Bilgen et al [8] stated that three-dimensional laparoscopy was superior to two-dimensional laparoscopy in terms of cholecystectomy. These academic inconsistencies highlight the clinical significance of our meta-analysis.

Although our meta-analysis was rigorously designed and performed, there were still some limitations within. Firstly, the statistical heterogeneity could not be thoroughly eliminated despite that we had conducted considerable amount of subgroup analyses. This is probably because that there is currently lacking of operative standards of three-dimensional laparoscopy. Different norms lead to inconsistent results and varied conclusions. Besides, more potential confounding elements are also needed to be explored and considered. Secondly, the number of included studies for secondary endpoints was not adequate to make a convincing conclusion. Well-prepared investigations are always needed for the future updates and supplements.

Taken together, through our systematic review and meta-analysis, we believe that three-dimensional laparoscopy is a preferably technical option against two-dimensional laparoscopy due to its better surgical efficacy. Thus a wider clinical application of 3D laparoscopy is strongly recommended.

## MATERIALS AND METHODS

This systematic study was classically performed as Cochrane Collaboration recommended. Each step of pooled analysis was independently conducted by two investigators, while any disagreement was settled by mutual discussion.

### Literature retrieval

Databases of PubMed, Web of Science, EMBASE and Cochrane Library were carefully screened using search term of “three dimensional laparoscopic”. Abstracts, full-texts and reference lists were thoroughly examined to avoid unnecessary omission during retrieval process.

### Selection criteria

Inclusion criteria: 1. Formally published studies until February 2016; 2.Comparing the clinical efficacy between 3D and 2D laparoscopy; 3. Adequate and accessible data of target endpoints;

Exclusion criteria: 1. Overlapped or duplicated publications; 2. Insufficient scale of sample-size (< 10); 3. Inappropriate article types such as Reviews and Case reports;

### Data extraction

A standardized form was designed to facilitate the extraction process. Original data of demographic elements (Study name; Country; Trial type; Surgical type; Group; Sample-size; Age; Sex), primary endpoints (Surgical time; Blood loss; Perioperative complications; Hospital stay; Conversion rate) and secondary endpoints (Drainage volume; Drainage time; Numbers of retrieved lymph nodes; Hospital expenses; Anastomosis time in prostatectomy; 6-month continence rate) were retrieved from tables, main text, figures and supplementary information among included studies. Continuous variables were rounded to one decimal place.

### Methodological quality appraisal

Observational investigations were assessed by Newcastle-Ottawa Scale (NOS). The entire scale was constituted by three categories including selection, comparability and outcome, with a maximum score of nine. Studies graded with six or more scores were identified as high-quality in methodology.

A Revised Jadad's Scale was employed to evaluate randomized trials. Randomization, allocation concealment, blindness and withdrawal were four scoring items, with a full credit of seven. Studies rated with four marks or more were recognized as high-quality in methodology.

### Statistical analysis

Review Manager 5.3 served as a statistical platform. Dichotomous and continuous variables were respectively analyzed by odds ratio (OR) and weighted mean difference (WMD). If the original data were inappropriately provided, median was taken for mean while standard deviation was estimated from range, interquartile range or 95% confidence interval, according to the instructions from Cochrane Handbook. Statistical heterogeneity was denoted by the degree of inconsistency (I^2^). Fixed-effects model was preferred when I^2^ value was less than 25%, otherwise a random-effects model was chosen. Primary endpoints were additionally divided into multiple subgroups based on different study types and surgical types. Sensitivity analysis was conducted by removing low-quality studies and interchanging calculation models (fixed-effects and random-effects), in order to observe the outcome stability. Publication bias was analyzed by Begg's test, Egger's test and Trim-and-Fill method. Statistical significance was indicated by *P* < 0.05.

## SUPPLEMENTARY MATERIAL


