# Supplementary material for: Two-dimensional versus three-dimensional laparoscopy in surgical efficacy: a systematic review and meta-analysis
Source: Oncotarget. 2016 Jul 29;7(43):70979–90. doi: 10.18632/oncotarget.10916 (PMC5342603; doi:10.18632/oncotarget.10916)
Supplement: Supplementary file 1 [file oncotarget-07-70979-s001.pdf]

Two-dimensional *versus* three-dimensional laparoscopy in surgical efficacy: a systematic review and meta-analysis

Supplementary Material

	2D		3D			Odds Ratio
Study or Subgroup	Events	Total	Events	Total	Weight	M-H, Fixed, 95% CI
**3.1.1 Retrospective**						
Agrusa 2015	2	26	0	13	1.6%	2.76 [0.12, 61.66]
Aykan 2014	3	66	0	29	1.7%	3.25 [0.16, 65.00]
Bove 2015	15	43	8	43	13.7%	2.34 [0.87, 6.32]
Chen 2015	4	34	1	26	2.6%	3.33 [0.35, 31.77]
Curro 2016	0	25	1	25	3.9%	0.32 [0.01, 8.25]
Usta 2014	8	91	4	56	11.9%	1.25 [0.36, 4.37]
Velayutham 2016	13	40	6	20	14.2%	1.12 [0.35, 3.59]
Xu 2014	1	15	0	16	1.2%	3.41 [0.13, 90.49]
Xu 2015	1	32	0	18	1.6%	1.76 [0.07, 45.52]
Zeng 2016	3	43	3	46	7.1%	1.07 [0.20, 5.64]
**Subtotal (95% CI)**		**415**		**292**	**59.6%**	**1.64 [0.97, 2.77]**
Total events	50		23			
Heterogeneity: Chi² = 3.18, df = 9 (P = 0.96); I² = 0%						
Test for overall effect: Z = 1.84 (P = 0.07)						
**3.1.2 Randomized**						
Chen 2016	0	20	0	25		Not estimable
Curro 2015-1	0	20	0	20		Not estimable
Curro 2015-2	0	20	0	20		Not estimable
Hou 2015	11	76	10	78	22.3%	1.15 [0.46, 2.89]
Kinoshita 2015	6	57	4	59	9.3%	1.62 [0.43, 6.06]
Navarra 2015	0	35	0	35		Not estimable
Ruan 2015	7	45	4	45	8.9%	1.89 [0.51, 6.97]
**Subtotal (95% CI)**		**273**		**282**	**40.4%**	**1.42 [0.74, 2.72]**
Total events	24		18			
Heterogeneity: Chi² = 0.42, df = 2 (P = 0.81); I² = 0%						
Test for overall effect: Z = 1.06 (P = 0.29)						
**Total (95% CI)**		**688**		**574**	**100.0%**	**1.55 [1.03, 2.33]**
Total events	74		41			
Heterogeneity: Chi² = 3.70, df = 12 (P = 0.99); I² = 0%						
Test for overall effect: Z = 2.10 (P = 0.04)						
Test for subgroup differences: Chi² = 0.11, df = 1 (P = 0.74), I² = 0%						

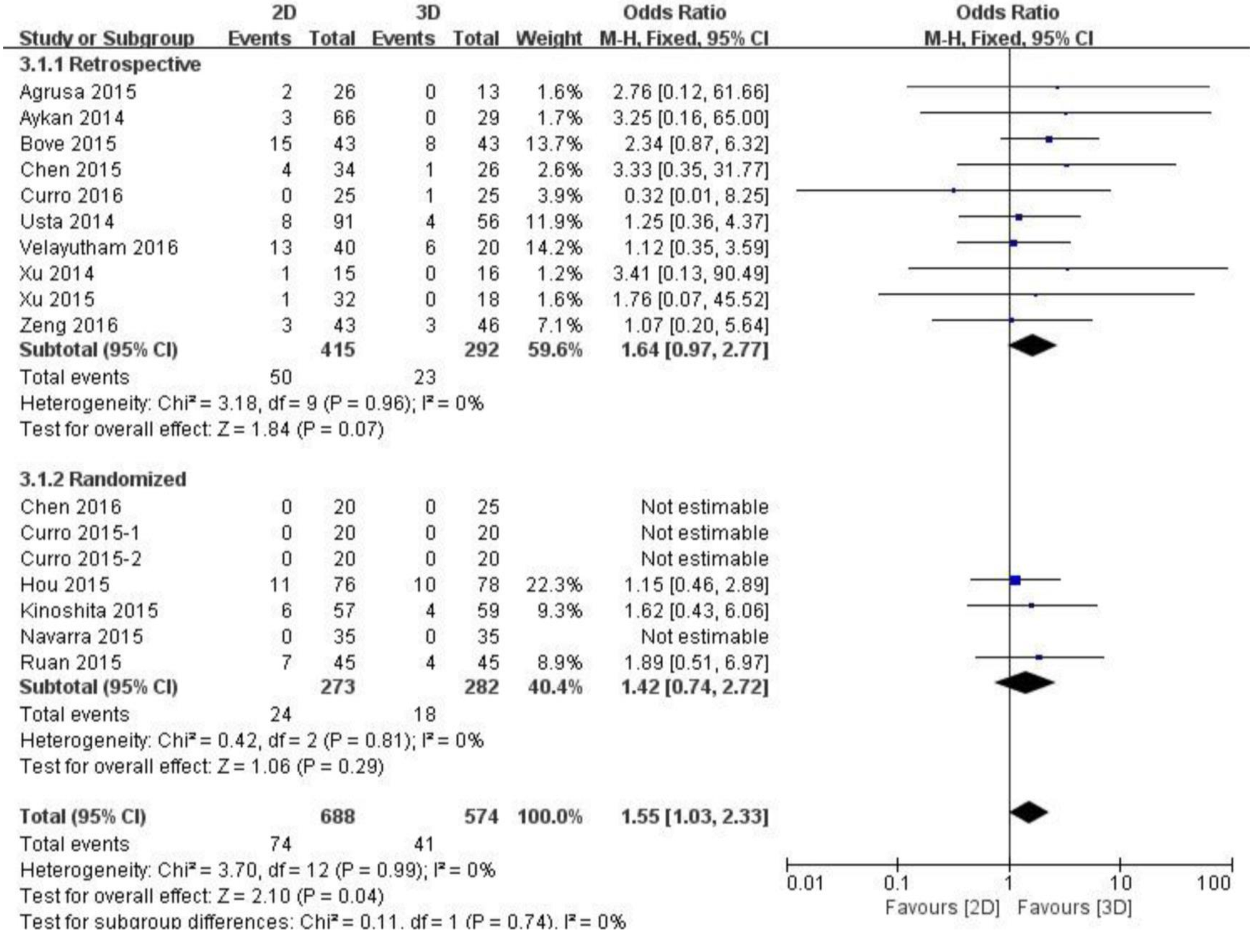

Supplementary Figure S1-The comparison of perioperative complications according to different study types

Study or Subgroup	2D Events	2D Total	3D Events	3D Total	Weight	Odds Ratio M-H, Fixed, 95% CI
**3.2.1 Cholecystectomy**						
Navarra 2015	0	35	0	35		Not estimable
Zeng 2016	3	43	3	46	7.1%	1.07 [0.20, 5.64]
**Subtotal (95% CI)**		**78**		**81**	**7.1%**	**1.07 [0.20, 5.64]**
Total events	3		3			
Heterogeneity: Not applicable						
Test for overall effect: Z = 0.09 (P = 0.93)						
**3.2.2 Prostatectomy**						
Aykan 2014	3	66	0	29	1.7%	3.25 [0.16, 65.00]
Bove 2015	15	43	8	43	13.7%	2.34 [0.87, 6.32]
Kinoshita 2015	6	57	4	59	9.3%	1.62 [0.43, 6.06]
Xu 2015	1	32	0	18	1.6%	1.76 [0.07, 45.52]
**Subtotal (95% CI)**		**198**		**149**	**26.3%**	**2.11 [1.00, 4.44]**
Total events	25		12			
Heterogeneity: Chi² = 0.29, df = 3 (P = 0.96); I² = 0%						
Test for overall effect: Z = 1.97 (P = 0.05)						
**3.2.3 Urological operations**						
Agrusa 2015	2	26	0	13	1.6%	2.76 [0.12, 61.66]
Chen 2016	0	20	0	25		Not estimable
Ruan 2015	7	45	4	45	8.9%	1.89 [0.51, 6.97]
Xu 2014	1	15	0	16	1.2%	3.41 [0.13, 90.49]
**Subtotal (95% CI)**		**106**		**99**	**11.6%**	**2.16 [0.70, 6.63]**
Total events	10		4			
Heterogeneity: Chi² = 0.14, df = 2 (P = 0.93); I² = 0%						
Test for overall effect: Z = 1.34 (P = 0.18)						
**3.2.4 Digestive operations**						
Curro 2015-1	0	20	0	20		Not estimable
Curro 2015-2	0	20	0	20		Not estimable
Curro 2016	0	25	1	25	3.9%	0.32 [0.01, 8.25]
Hou 2015	11	76	10	78	22.3%	1.15 [0.46, 2.89]
Velayutham 2016	13	40	6	20	14.2%	1.12 [0.35, 3.59]
**Subtotal (95% CI)**		**181**		**163**	**40.4%**	**1.06 [0.53, 2.13]**
Total events	24		17			
Heterogeneity: Chi² = 0.56, df = 2 (P = 0.76); I² = 0%						
Test for overall effect: Z = 0.17 (P = 0.87)						
**3.2.5 Other types**						
Chen 2015	4	34	1	26	2.6%	3.33 [0.35, 31.77]
Usta 2014	8	91	4	56	11.9%	1.25 [0.36, 4.37]
**Subtotal (95% CI)**		**125**		**82**	**14.5%**	**1.63 [0.55, 4.79]**
Total events	12		5			
Heterogeneity: Chi² = 0.56, df = 1 (P = 0.46); I² = 0%						
Test for overall effect: Z = 0.89 (P = 0.37)						
**Total (95% CI)**		**688**		**574**	**100.0%**	**1.55 [1.03, 2.33]**
Total events	74		41			
Heterogeneity: Chi² = 3.70, df = 12 (P = 0.99); I² = 0%						
Test for overall effect: Z = 2.10 (P = 0.04)						
Test for subgroup differences: Chi² = 2.32, df = 4 (P = 0.68), I² = 0%						

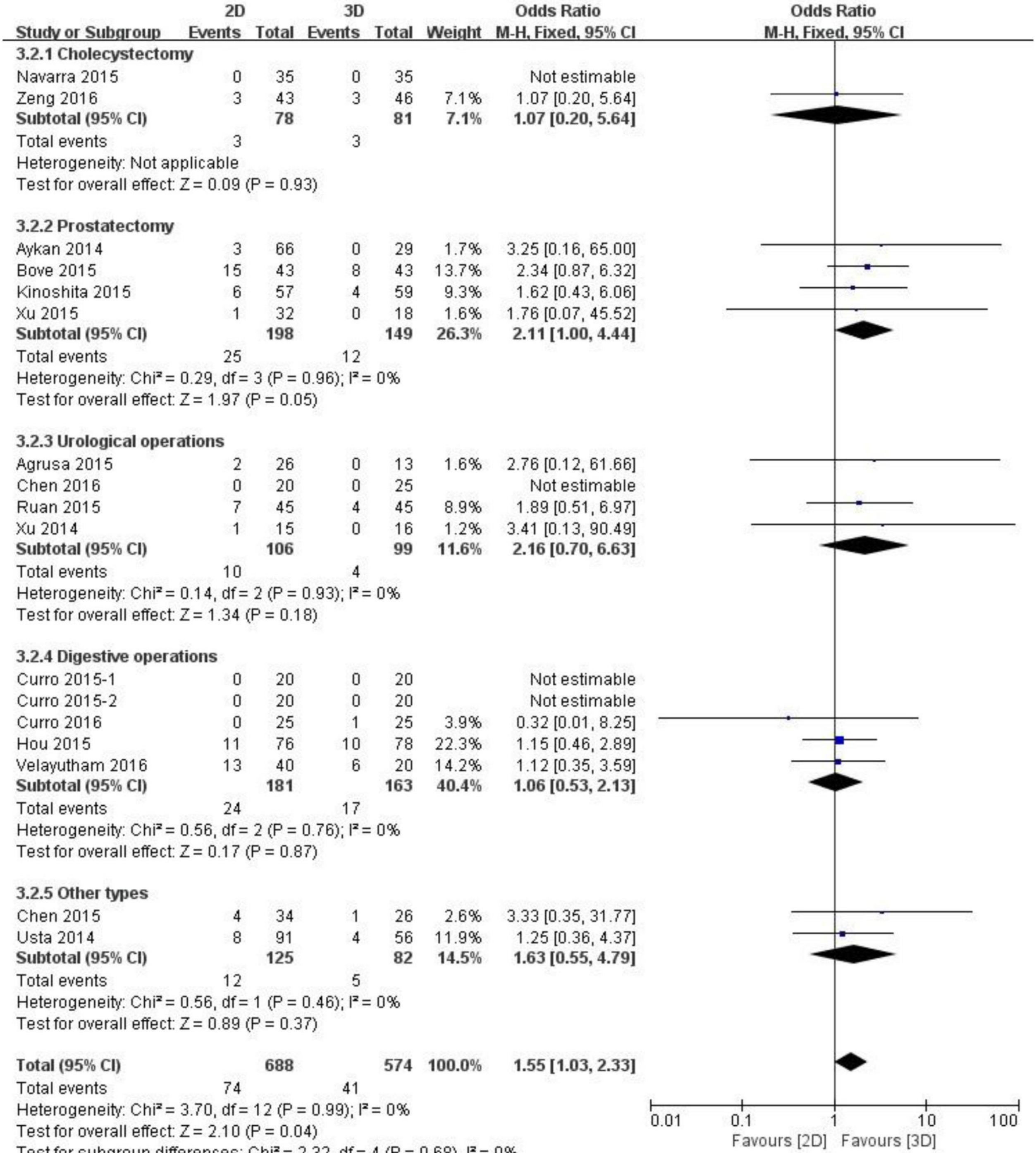

Supplementary Figure S2-The comparison of perioperative complications according to different surgical types

Study or Subgroup	2D Mean	2D SD	2D Total	3D Mean	3D SD	3D Total	Weight	Mean Difference IV, Random, 95% CI
**4.1.1 Retrospective**								
Bove 2015	7.6	7.2	43	5.5	7.2	43	2.1%	2.10 [-0.94, 5.14]
Usta 2014	3.3	1.4	91	3	1.4	56	25.2%	0.30 [-0.17, 0.77]
Velayutham 2016	8	5	40	9	7.8	20	1.4%	-1.00 [-4.75, 2.75]
Xu 2014	7.5	0.6	15	7.3	0.7	16	25.4%	0.20 [-0.26, 0.66]
Xu 2015	20.2	5.5	32	14.4	7.2	18	1.4%	5.80 [1.97, 9.63]
Zeng 2016	5.3	1.2	43	5.1	2.1	46	18.8%	0.20 [-0.50, 0.90]
Zou 2014	4	1.1	30	3.1	1	30	23.3%	0.90 [0.37, 1.43]
**Subtotal (95% CI)**			**294**			**229**	**97.6%**	**0.50 [0.02, 0.99]**
Heterogeneity: Tau² = 0.18; Chi² = 13.93, df = 6 (P = 0.03); I² = 57%								
Test for overall effect: Z = 2.03 (P = 0.04)								
**4.1.2 Randomized**								
Hou 2015	12.6	8.8	76	11.8	9.3	78	2.4%	0.80 [-2.06, 3.66]
**Subtotal (95% CI)**			**76**			**78**	**2.4%**	**0.80 [-2.06, 3.66]**
Heterogeneity: Not applicable								
Test for overall effect: Z = 0.55 (P = 0.58)								
**Total (95% CI)**			**370**			**307**	**100.0%**	**0.50 [0.04, 0.96]**
Heterogeneity: Tau² = 0.16; Chi² = 13.99, df = 7 (P = 0.05); I² = 50%								
Test for overall effect: Z = 2.15 (P = 0.03)								
Test for subgroup differences: Chi² = 0.04, df = 1 (P = 0.84), I² = 0%								

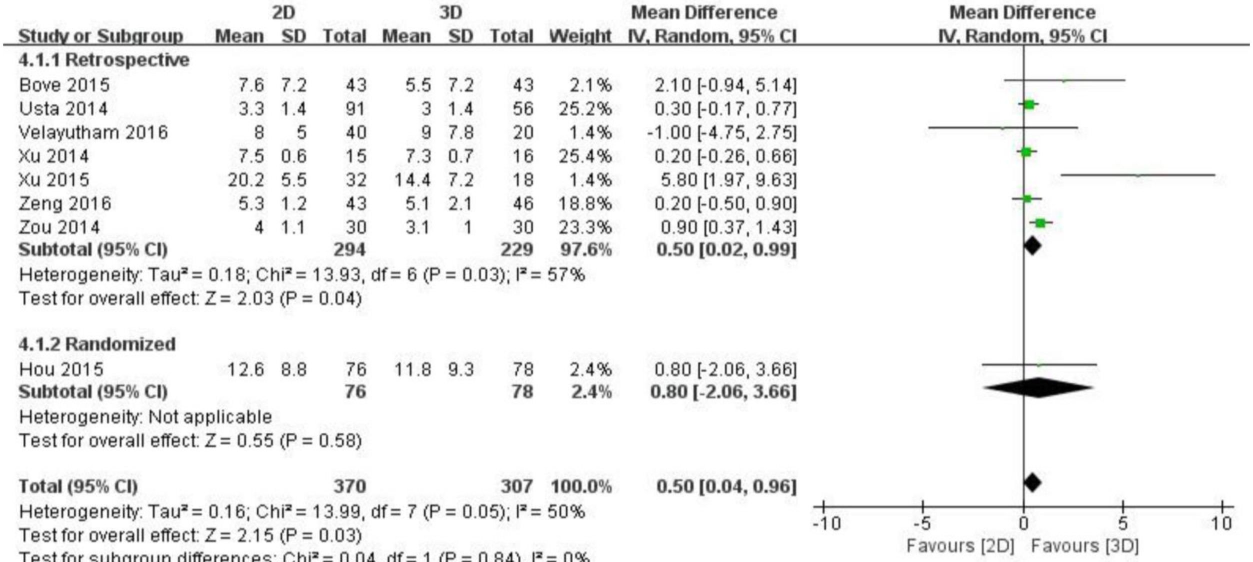

Supplementary Figure S3-The comparison of hospital stay according to different study types.

Study or Subgroup	2D Mean	2D SD	2D Total	3D Mean	3D SD	3D Total	Weight	Mean Difference IV, Random, 95% CI
**4.2.1 Cholecystectomy**								
Zeng 2016	5.3	1.2	43	5.1	2.1	46	18.8%	0.20 [-0.50, 0.90]
**Subtotal (95% CI)**			**43**			**46**	**18.8%**	**0.20 [-0.50, 0.90]**
Heterogeneity: Not applicable								
Test for overall effect: Z = 0.56 (P = 0.58)								
**4.2.2 Prostatectomy**								
Bove 2015	7.6	7.2	43	5.5	7.2	43	2.1%	2.10 [-0.94, 5.14]
Xu 2015	20.2	5.5	32	14.4	7.2	18	1.4%	5.80 [1.97, 9.63]
**Subtotal (95% CI)**			**75**			**61**	**3.5%**	**3.76 [0.15, 7.37]**
Heterogeneity: Tau² = 3.73; Chi² = 2.20, df = 1 (P = 0.14); I² = 54%								
Test for overall effect: Z = 2.04 (P = 0.04)								
**4.2.3 Urological operations**								
Xu 2014	7.5	0.6	15	7.3	0.7	16	25.4%	0.20 [-0.26, 0.66]
**Subtotal (95% CI)**			**15**			**16**	**25.4%**	**0.20 [-0.26, 0.66]**
Heterogeneity: Not applicable								
Test for overall effect: Z = 0.86 (P = 0.39)								
**4.2.4 Digestive operations**								
Hou 2015	12.6	8.8	76	11.8	9.3	78	2.4%	0.80 [-2.06, 3.66]
Velayutham 2016	8	5	40	9	7.8	20	1.4%	-1.00 [-4.75, 2.75]
**Subtotal (95% CI)**			**116**			**98**	**3.8%**	**0.14 [-2.14, 2.41]**
Heterogeneity: Tau² = 0.00; Chi² = 0.56, df = 1 (P = 0.45); I² = 0%								
Test for overall effect: Z = 0.12 (P = 0.90)								
**4.2.5 Other types**								
Usta 2014	3.3	1.4	91	3	1.4	56	25.2%	0.30 [-0.17, 0.77]
Zou 2014	4	1.1	30	3.1	1	30	23.3%	0.90 [0.37, 1.43]
**Subtotal (95% CI)**			**121**			**86**	**48.5%**	**0.59 [-0.00, 1.17]**
Heterogeneity: Tau² = 0.11; Chi² = 2.76, df = 1 (P = 0.10); I² = 64%								
Test for overall effect: Z = 1.95 (P = 0.05)								
**Total (95% CI)**			**370**			**307**	**100.0%**	**0.50 [0.04, 0.96]**
Heterogeneity: Tau² = 0.16; Chi² = 13.99, df = 7 (P = 0.05); I² = 50%								
Test for overall effect: Z = 2.15 (P = 0.03)								
Test for subgroup differences: Chi² = 4.66, df = 4 (P = 0.32), I² = 14.3%								

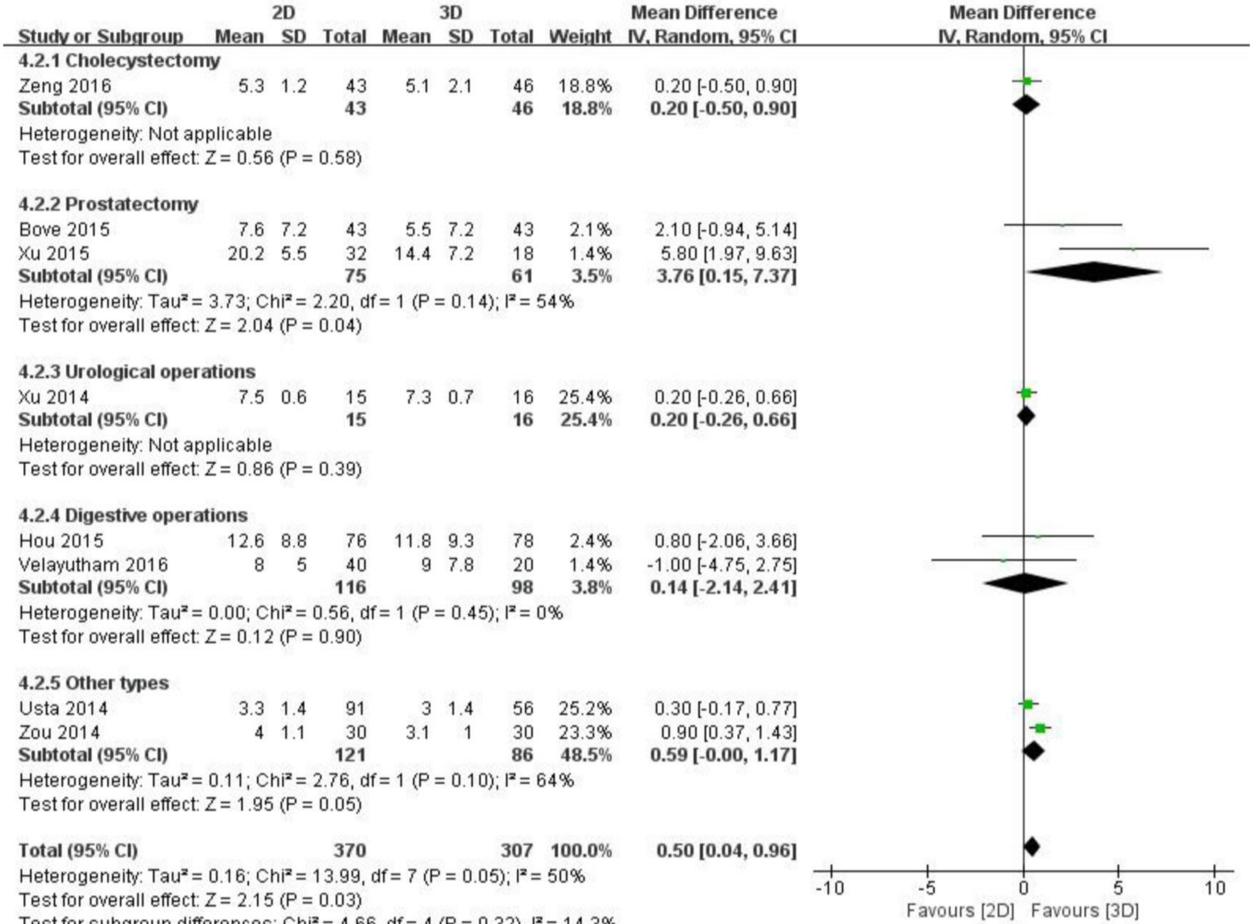

Supplementary Figure S4-The comparison of hospital stay according to different surgical types.

Study or Subgroup	2D Events	2D Total	3D Events	3D Total	Weight	Odds Ratio M-H, Fixed, 95% CI
**5.1.1 Retrospective**						
Agrusa 2015	0	26	0	13		Not estimable
Aykan 2014	0	66	0	29		Not estimable
Bove 2015	0	43	0	43		Not estimable
Chen 2015	1	34	0	26	12.1%	2.37 [0.09, 60.65]
Curro 2016	0	25	0	25		Not estimable
Velayutham 2016	1	40	1	20	29.1%	0.49 [0.03, 8.22]
Xu 2014	0	15	0	16		Not estimable
Xu 2015	0	32	0	18		Not estimable
Zeng 2016	4	43	3	46	58.8%	1.47 [0.31, 6.98]
Zou 2014	0	30	0	30		Not estimable
**Subtotal (95% CI)**		**354**		**266**	**100.0%**	**1.29 [0.37, 4.46]**
Total events	6		4			
Heterogeneity: Chi² = 0.62, df = 2 (P = 0.73); I² = 0%						
Test for overall effect: Z = 0.41 (P = 0.68)						
**5.1.2 Randomized**						
Chen 2016	0	20	0	25		Not estimable
Curro 2015-1	0	20	0	20		Not estimable
Curro 2015-2	0	20	0	20		Not estimable
Hanna 1998	0	30	0	30		Not estimable
Hou 2015	0	76	0	78		Not estimable
Navarra 2015	0	35	0	35		Not estimable
Ruan 2015	0	45	0	45		Not estimable
**Subtotal (95% CI)**		**246**		**253**		**Not estimable**
Total events	0		0			
Heterogeneity: Not applicable						
Test for overall effect: Not applicable						
**Total (95% CI)**		**600**		**519**	**100.0%**	**1.29 [0.37, 4.46]**
Total events	6		4			
Heterogeneity: Chi² = 0.62, df = 2 (P = 0.73); I² = 0%						
Test for overall effect: Z = 0.41 (P = 0.68)						
Test for subgroup differences: Not applicable						

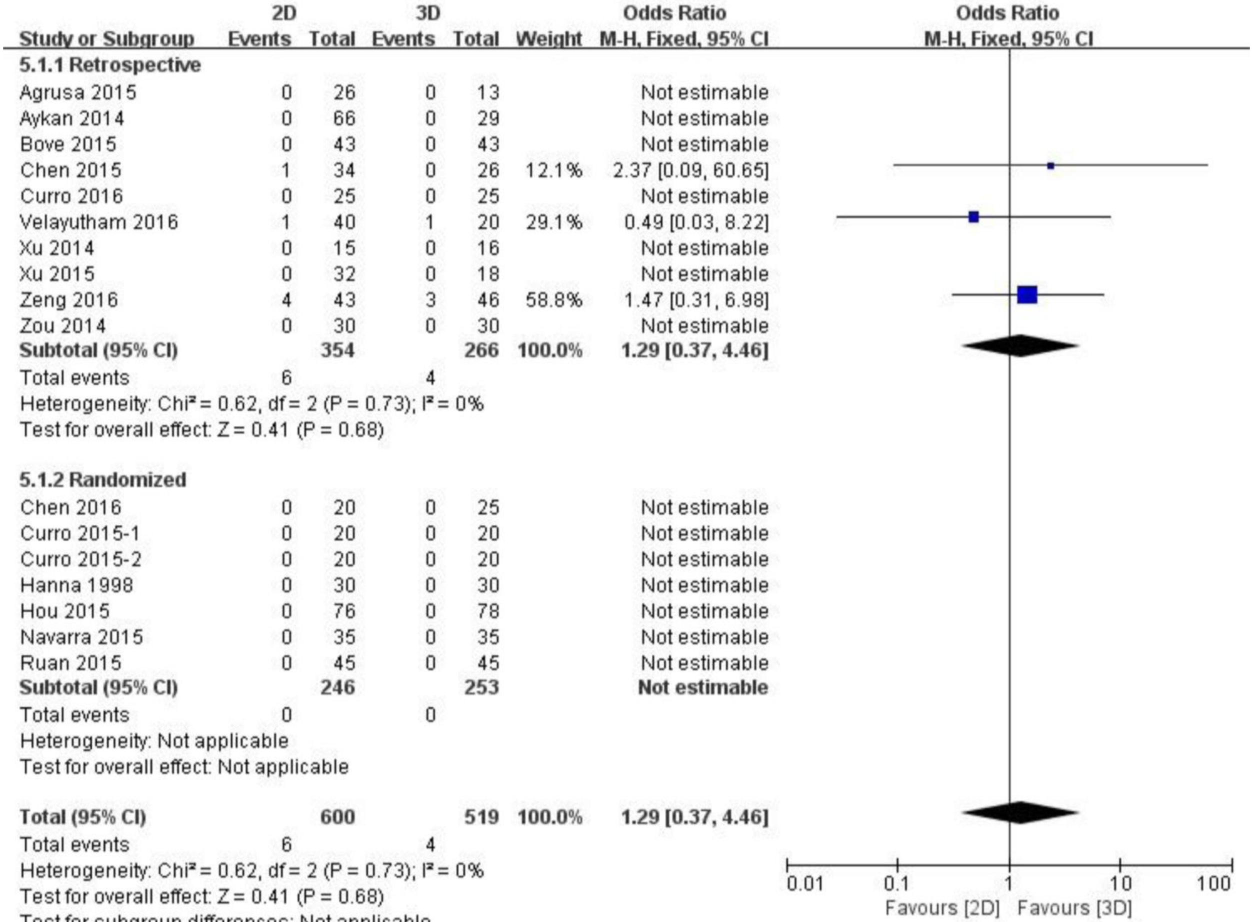

Supplementary Figure S5-The comparison of conversion rate according to different study types

Study or Subgroup	2D Events	2D Total	3D Events	3D Total	Weight	Odds Ratio M-H, Fixed, 95% CI
**5.2.1 Cholecystectomy**						
Navarra 2015	0	35	0	35		Not estimable
Zeng 2016	4	43	3	46	58.8%	1.47 [0.31, 6.98]
**Subtotal (95% CI)**		**78**		**81**	**58.8%**	**1.47 [0.31, 6.98]**
Total events	4		3			
Heterogeneity: Not applicable						
Test for overall effect: Z = 0.48 (P = 0.63)						
**5.2.2 Prostatectomy**						
Aykan 2014	0	66	0	29		Not estimable
Bove 2015	0	43	0	43		Not estimable
Xu 2015	0	32	0	18		Not estimable
**Subtotal (95% CI)**		**141**		**90**		**Not estimable**
Total events	0		0			
Heterogeneity: Not applicable						
Test for overall effect: Not applicable						
**5.2.3 Urological operations**						
Agrusa 2015	0	26	0	13		Not estimable
Chen 2016	0	20	0	25		Not estimable
Ruan 2015	0	45	0	45		Not estimable
Xu 2014	0	15	0	16		Not estimable
**Subtotal (95% CI)**		**106**		**99**		**Not estimable**
Total events	0		0			
Heterogeneity: Not applicable						
Test for overall effect: Not applicable						
**5.2.4 Digestive operations**						
Curro 2015-1	0	20	0	20		Not estimable
Curro 2015-2	0	20	0	20		Not estimable
Curro 2016	0	25	0	25		Not estimable
Hou 2015	0	76	0	78		Not estimable
Velayutham 2016	1	40	1	20	29.1%	0.49 [0.03, 8.22]
**Subtotal (95% CI)**		**181**		**163**	**29.1%**	**0.49 [0.03, 8.22]**
Total events	1		1			
Heterogeneity: Not applicable						
Test for overall effect: Z = 0.50 (P = 0.62)						
**5.2.5 Other types**						
Chen 2015	1	34	0	26	12.1%	2.37 [0.09, 60.65]
Zou 2014	0	30	0	30		Not estimable
**Subtotal (95% CI)**		**64**		**56**	**12.1%**	**2.37 [0.09, 60.65]**
Total events	1		0			
Heterogeneity: Not applicable						
Test for overall effect: Z = 0.52 (P = 0.60)						
**Total (95% CI)**		**570**		**489**	**100.0%**	**1.29 [0.37, 4.46]**
Total events	6		4			
Heterogeneity: Chi² = 0.62, df = 2 (P = 0.73); I² = 0%						
Test for overall effect: Z = 0.41 (P = 0.68)						
Test for subgroup differences: Chi² = 0.62, df = 2 (P = 0.73), I² = 0%						

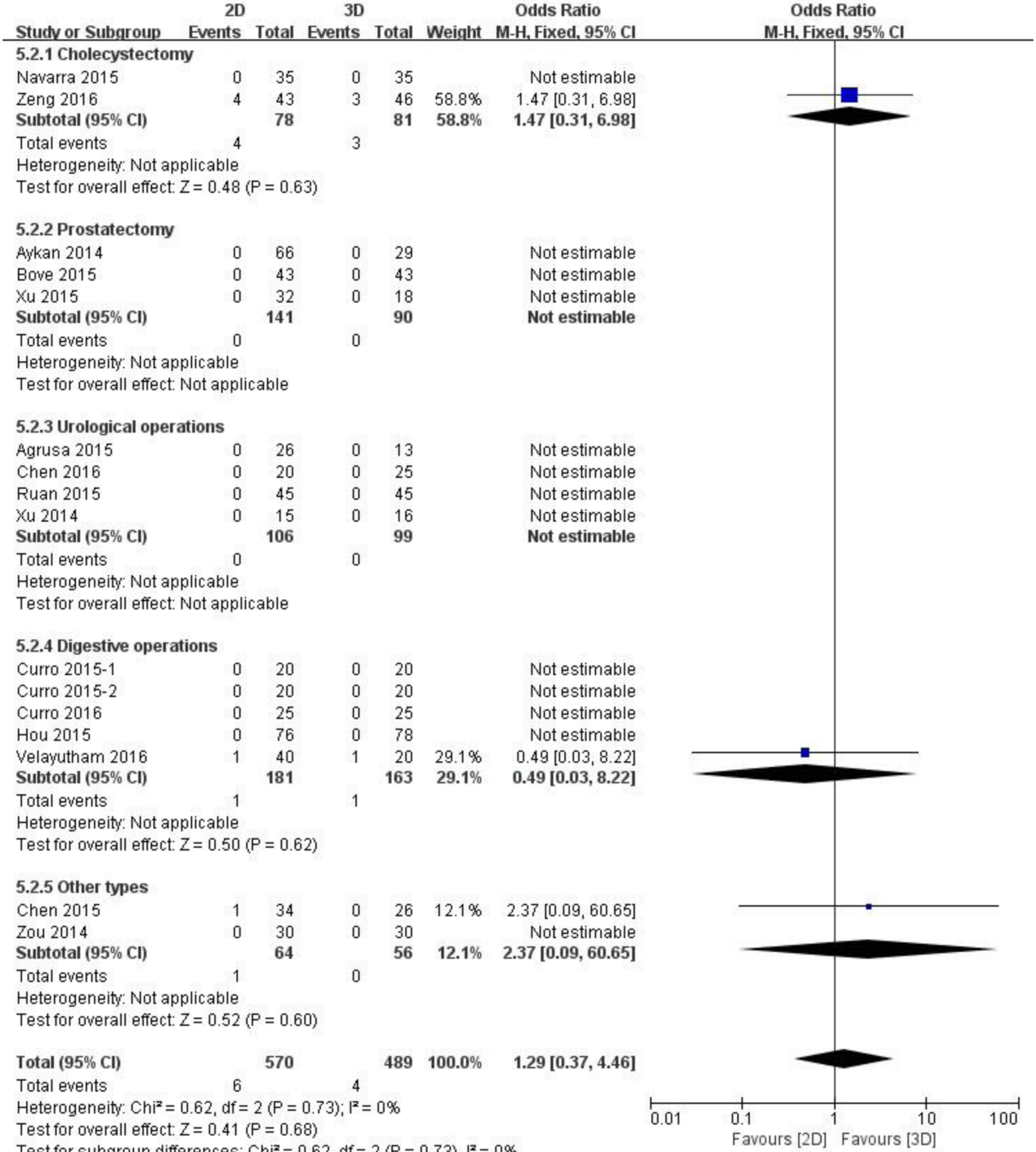

Supplementary Figure S6-The comparison of conversion rate according to different surgical types

Study or Subgroup	2D Mean	2D SD	2D Total	3D Mean	3D SD	3D Total	Weight	Mean Difference IV, Fixed, 95% CI
Chen 2015	101.5	52.8	34	119.5	53.5	26	2.4%	-18.00 [-45.16, 9.16]
Hou 2015	485	107.4	76	470	93.4	78	1.7%	15.00 [-16.82, 46.82]
Zou 2014	47.1	6.3	30	46.2	10.1	30	95.9%	0.90 [-3.36, 5.16]
**Total (95% CI)**			**140**			**134**	**100.0%**	**0.70 [-3.48, 4.87]**

Heterogeneity: Chi² = 2.60, df = 2 (P = 0.27); I² = 23%
Test for overall effect: Z = 0.33 (P = 0.74)

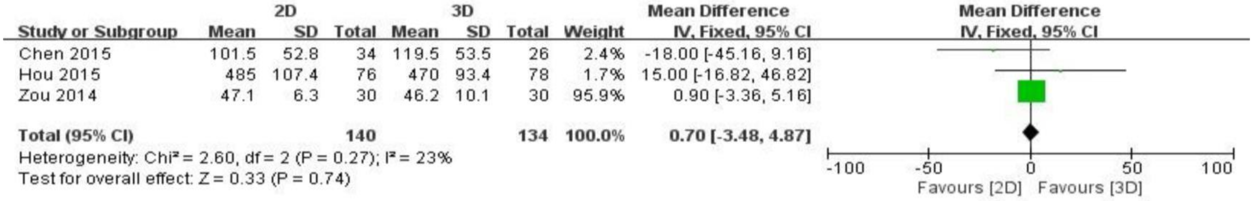

Supplementary Figure S7-The comparison of drainage volume between 2D and 3D laparoscopy

Study or Subgroup	2D Mean	2D SD	2D Total	3D Mean	3D SD	3D Total	Weight	Mean Difference IV, Random, 95% CI
Bove 2015	4.5	16.1	43	4.9	16.1	43	0.8%	-0.40 [-7.21, 6.41]
Chen 2015	3.3	0.6	34	3.5	0.7	26	38.8%	-0.20 [-0.54, 0.14]
Hou 2015	2.9	0.6	76	2.7	0.8	78	41.7%	0.20 [-0.02, 0.42]
Xu 2015	7.1	1.1	32	5.3	2.1	18	18.8%	1.80 [0.76, 2.84]
**Total (95% CI)**			**185**			**165**	**100.0%**	**0.34 [-0.25, 0.94]**

Heterogeneity: Tau² = 0.21; Chi² = 13.96, df = 3 (P = 0.003); I² = 79%
Test for overall effect: Z = 1.12 (P = 0.26)

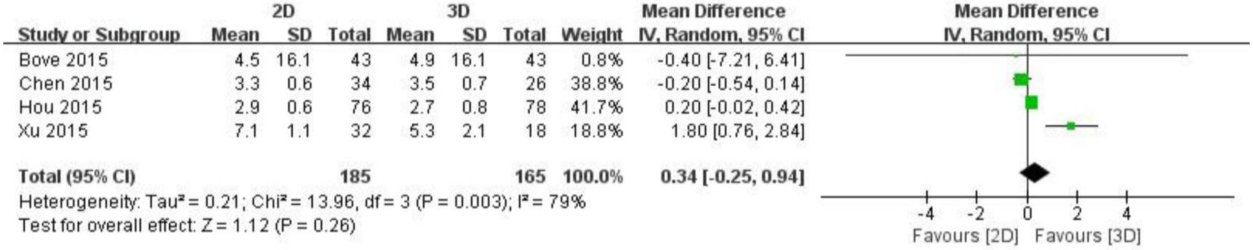

Supplementary Figure S8-The comparison of drainage time between 2D and 3D laparoscopy

Study or Subgroup	2D Mean	2D SD	2D Total	3D Mean	3D SD	3D Total	Weight	Mean Difference IV, Random, 95% CI
Ji 2014	21	9	20	24	6	16	33.7%	-3.00 [-7.92, 1.92]
Hou 2015	18	3.3	76	17	8.6	78	66.3%	1.00 [-1.05, 3.05]
**Total (95% CI)**			**96**			**94**	**100.0%**	**-0.35 [-4.06, 3.36]**

Heterogeneity: Tau² = 4.30; Chi² = 2.16, df = 1 (P = 0.14); I² = 54%
Test for overall effect: Z = 0.18 (P = 0.85)

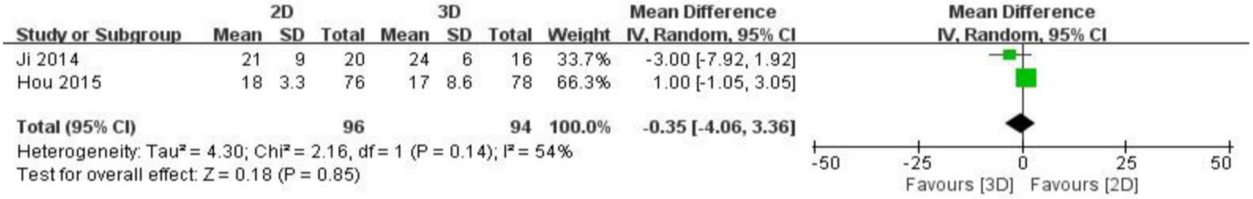

Supplementary Figure S9-The comparison of numbers of retrieved lymphnodes between 2D and 3D laparoscopy

Study or Subgroup	2D Mean	2D SD	2D Total	3D Mean	3D SD	3D Total	Weight	Mean Difference IV, Fixed, 95% CI
Chen 2014	7,100	1,300	40	7,500	1,600	40	65.5%	-400.00 [-1038.87, 238.87]
Xu 2014	25,426	2,626	15	25,687	3,032	16	6.7%	-261.00 [-2254.28, 1732.28]
Zou 2014	11,868	2,111	30	11,511	1,749	30	27.8%	357.00 [-623.98, 1337.98]
**Total (95% CI)**			**85**			**86**	**100.0%**	**-180.37 [-697.39, 336.66]**

Heterogeneity: Chi² = 1.61, df = 2 (P = 0.45); I² = 0%
Test for overall effect: Z = 0.68 (P = 0.49)

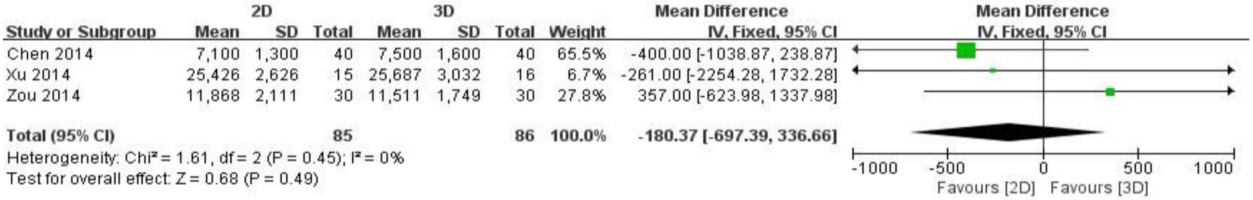

Supplementary Figure S10-The comparison of hospital expenses between 2D and 3D laparoscopy

Study or Subgroup	2D Mean	2D SD	2D Total	3D Mean	3D SD	3D Total	Weight	Mean Difference IV, Random, 95% CI
Aykan 2014	87	17	66	28	6	29	33.3%	59.00 [54.35, 63.65]
Bove 2015	32	6.3	43	24	8.8	43	33.4%	8.00 [4.77, 11.23]
Kinoshita 2015	30.1	13.5	57	26.7	8.7	59	33.3%	3.40 [-0.75, 7.55]
**Total (95% CI)**			**166**			**131**	**100.0%**	**23.44 [-8.32, 55.19]**

Heterogeneity: Tau² = 783.17; Chi² = 383.22, df = 2 (P < 0.00001); I² = 99%
Test for overall effect: Z = 1.45 (P = 0.15)

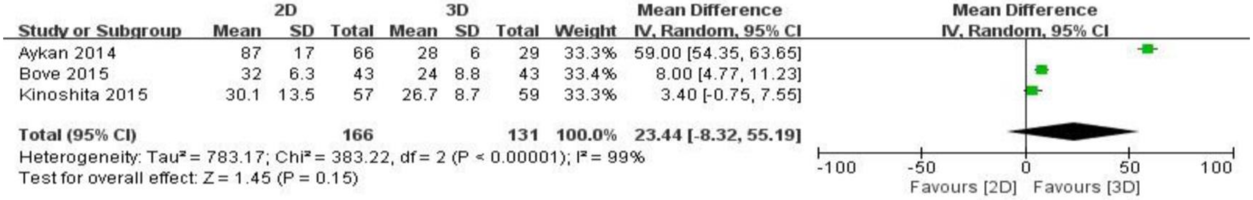

Supplementary Figure S11-The comparison of anatomosis time in prostatectomy between 2D and 3D laparoscopy

Study or Subgroup	2D Events	2D Total	3D Events	3D Total	Weight	Odds Ratio M-H, Fixed, 95% CI
Aykan 2014	64	66	28	29	16.0%	1.14 [0.10, 13.13]
Bove 2015	36	43	38	43	84.0%	0.68 [0.20, 2.33]
Xu 2015	32	32	18	18		Not estimable
**Total (95% CI)**		**141**		**90**	**100.0%**	**0.75 [0.25, 2.27]**
Total events	132		84			

Heterogeneity: Chi² = 0.14, df = 1 (P = 0.71); I² = 0%
Test for overall effect: Z = 0.51 (P = 0.61)

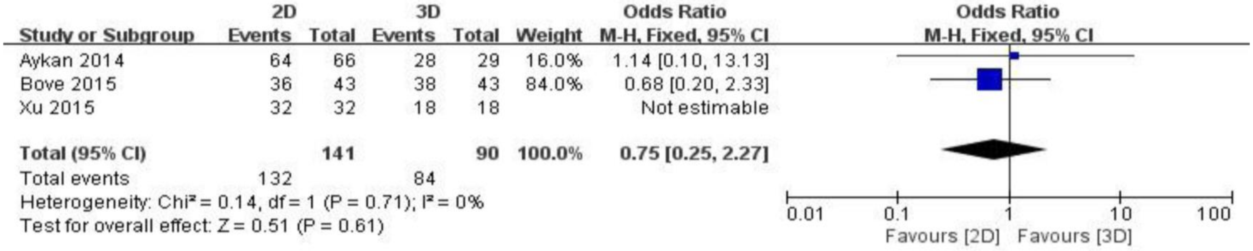

Supplementary Figure S12-The comparison of 6-month continence rate between 2D and 3D laparoscopy

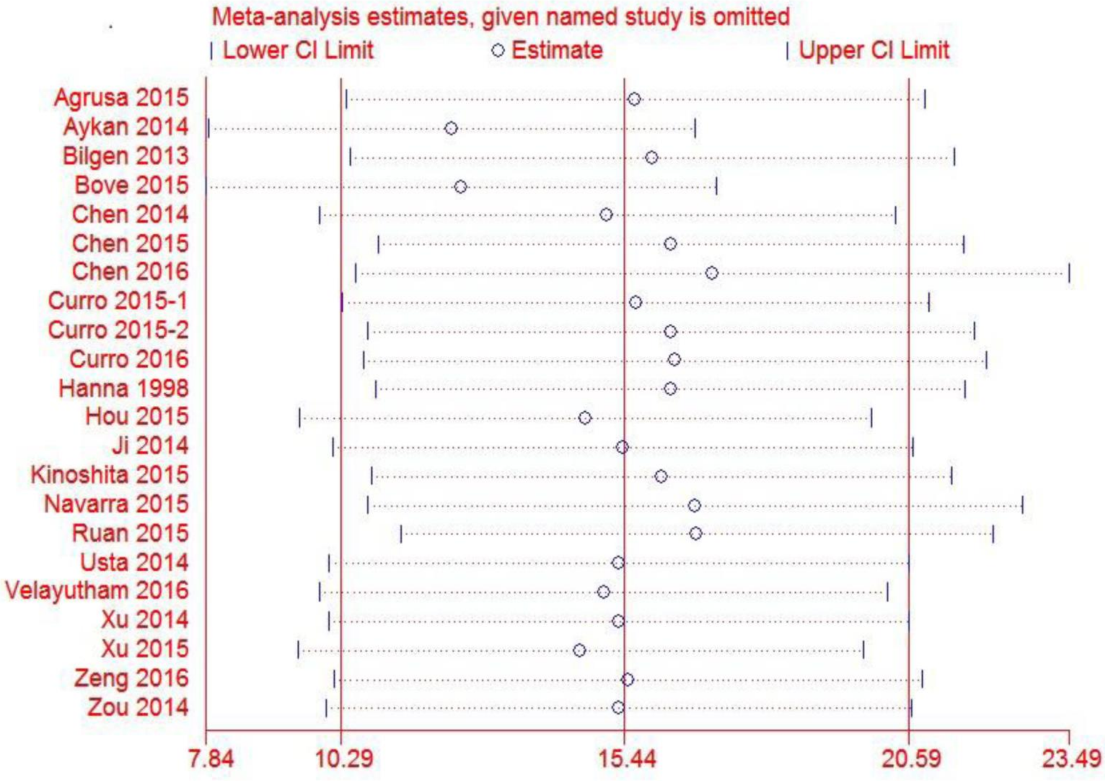

Supplementary Figure S13-Sensitivity analysis

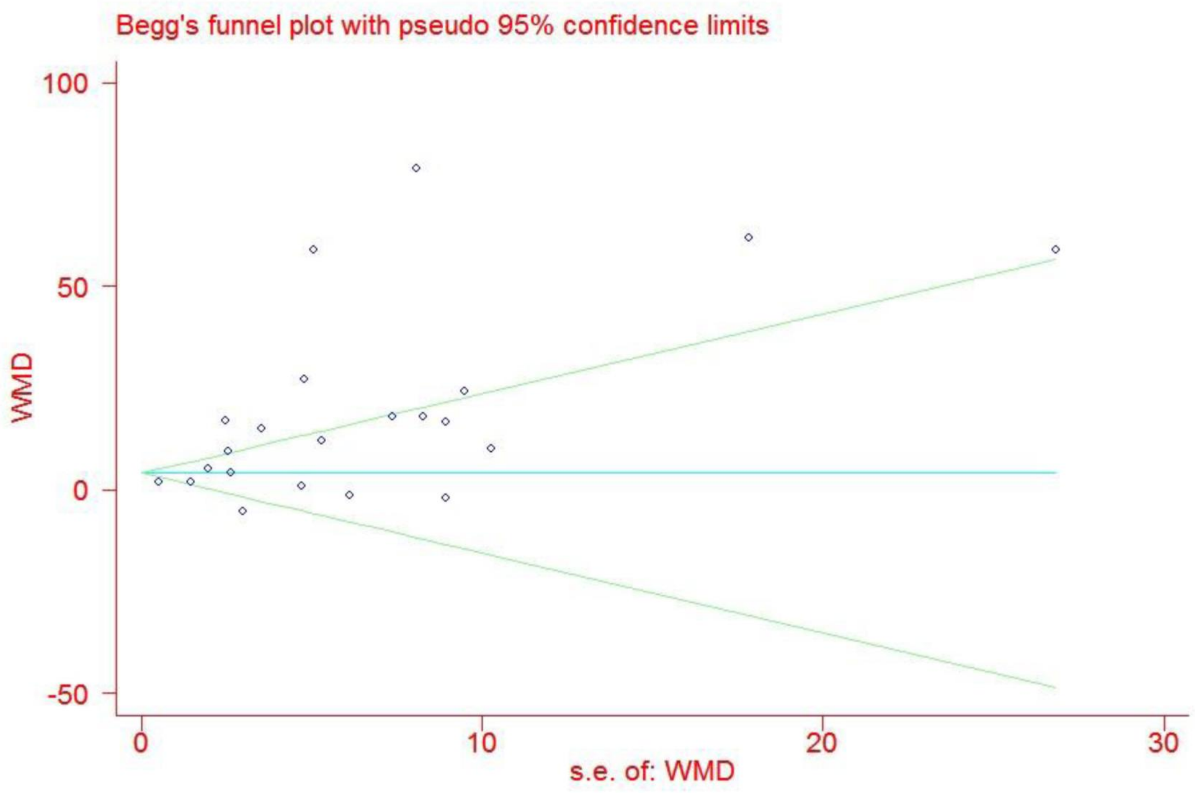

Supplementary Figure S14-Begg's test

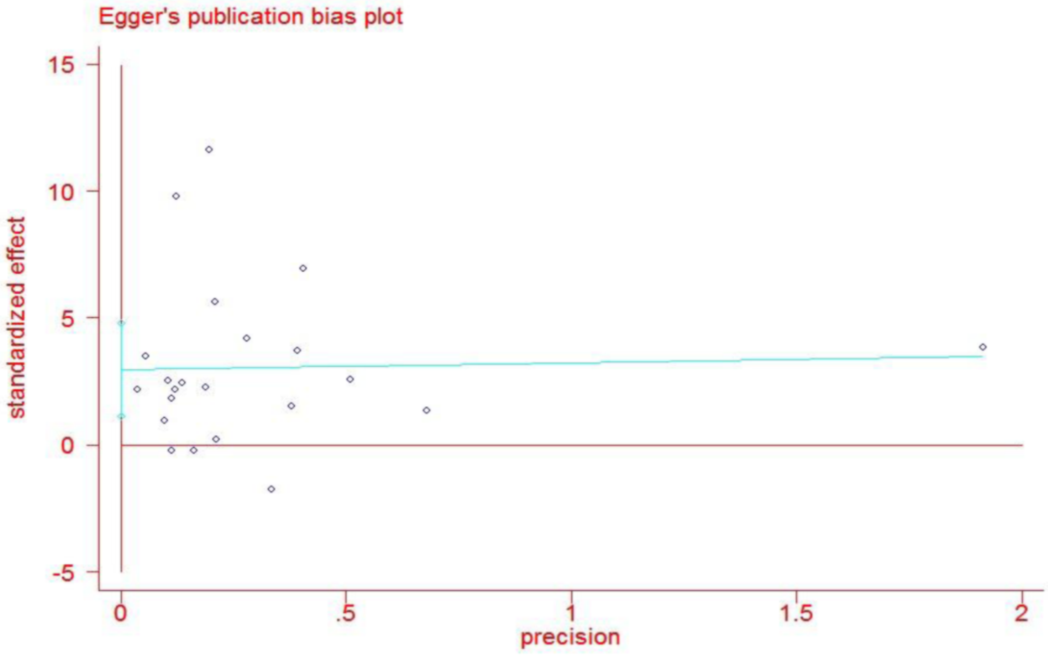

Supplementary Figure S15-Egger's test

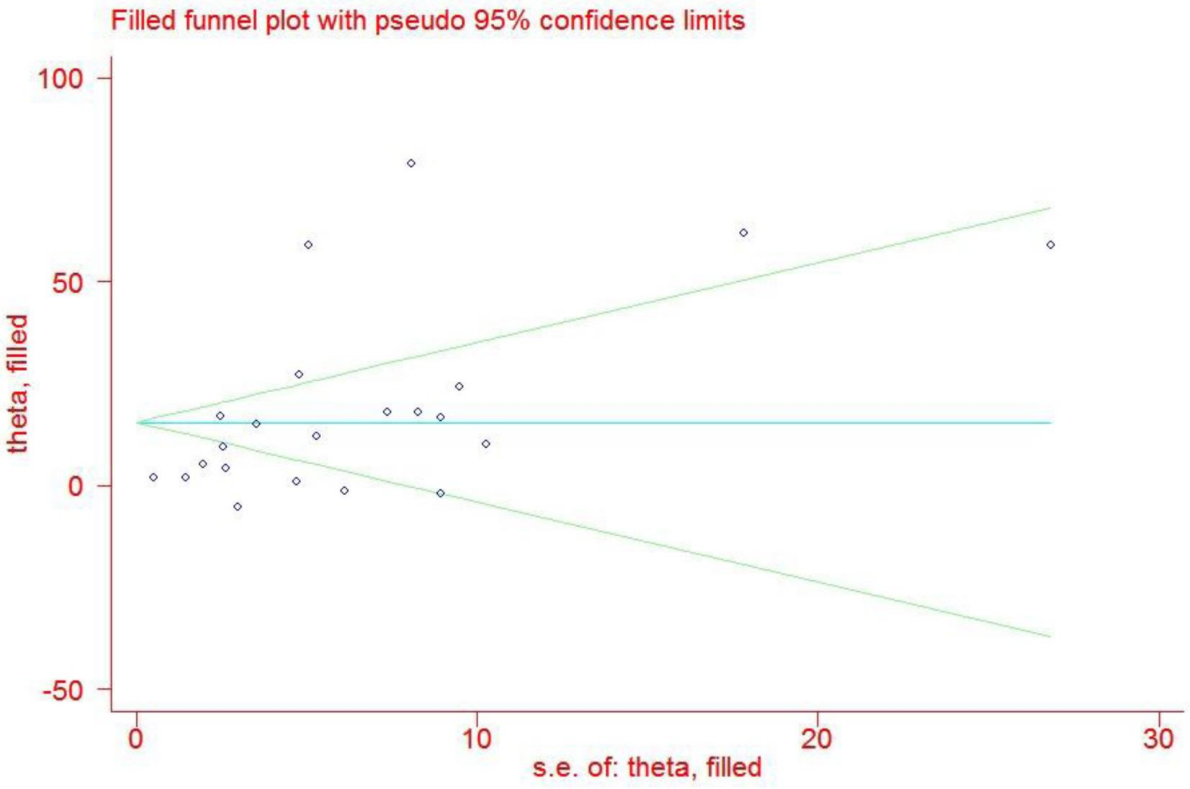

Supplementary Figure S16-Trim-and-Fill method

PRISMA 2009 Checklist

Section/Topic	#	Checklist Item	Reported on Page #
**TITLE**			
Title	1	Identify the report as a systematic review, meta-analysis, or both.	1
**ABSTRACT**			
Structured summary	2	Provide a structured summary including, as applicable: background; objectives; data sources; study eligibility criteria, participants, and interventions; study appraisal and synthesis methods; results; limitations; conclusions and implications of key findings; systematic review registration number.	2
**INTRODUCTION**			
Rationale	3	Describe the rationale for the review in the context of what is already known.	3
Objectives	4	Provide an explicit statement of questions being addressed with reference to participants, interventions, comparisons, outcomes, and study design (PICOS).	4
**METHODS**			
Protocol and registration	5	Indicate if a review protocol exists, if and where it can be accessed (e.g., Web address), and, if available, provide registration information including registration number.	12
Eligibility criteria	6	Specify study characteristics (e.g., PICOS, length of follow-up) and report characteristics (e.g., years considered, language, publication status) used as criteria for eligibility, giving rationale.	12
Information sources	7	Describe all information sources (e.g., databases with dates of coverage, contact with study authors to identify additional studies) in the search and date last searched.	12
Search	8	Present full electronic search strategy for at least one database, including any limits used, such that it could be repeated.	12
Study selection	9	State the process for selecting studies (i.e., screening, eligibility, included in systematic review, and, if applicable, included in the meta-analysis).	12
Data collection process	10	Describe method of data extraction from reports (e.g., piloted forms, independently, in duplicate) and any processes for obtaining and confirming data from investigators.	13
Data items	11	List and define all variables for which data were sought (e.g., PICOS, funding sources) and any assumptions and simplifications made.	13
Risk of bias in individual studies	12	Describe methods used for assessing risk of bias of individual studies (including specification of whether this was done at the study or outcome level), and how this information is to be used in any data synthesis.	13
Summary measures	13	State the principal summary measures (e.g., risk ratio, difference in means).	13-14
Synthesis of results	14	Describe the methods of handling data and combining results of studies, if done, including measures of consistency (e.g., I^2) for each meta-analysis.	14

PRISMA 2009 Checklist

Section/Topic	#	Checklist Item	Reported on Page #
Risk of bias across studies	15	Specify any assessment of risk of bias that may affect the cumulative evidence (e.g., publication bias, selective reporting within studies).	13
Additional analyses	16	Describe methods of additional analyses (e.g., sensitivity or subgroup analyses, meta-regression), if done, indicating which were pre-specified.	13
**RESULTS**			
Study selection	17	Give numbers of studies screened, assessed for eligibility, and included in the review, with reasons for exclusions at each stage, ideally with a flow diagram.	4
Study characteristics	18	For each study, present characteristics for which data were extracted (e.g., study size, PICOS, follow-up period) and provide the citations.	4
Risk of bias within studies	19	Present data on risk of bias of each study and, if available, any outcome level assessment (see item 12).	5
Results of individual studies	20	For all outcomes considered (benefits or harms), present, for each study: (a) simple summary data for each intervention group (b) effect estimates and confidence intervals, ideally with a forest plot.	5-8
Synthesis of results	21	Present the main results of the review. If meta-analyses done, include for each, confidence intervals and measures of consistency.	5-8
Risk of bias across studies	22	Present results of any assessment of risk of bias across studies (see Item 15).	8
Additional analysis	23	Give results of additional analyses, if done (e.g., sensitivity or subgroup analyses, meta-regression [see Item 16]).	9
**DISCUSSION**			
Summary of evidence	24	Summarize the main findings including the strength of evidence for each main outcome; consider their relevance to key groups (e.g., healthcare providers, users, and policy makers).	9-11
Limitations	25	Discuss limitations at study and outcome level (e.g., risk of bias), and at review-level (e.g., incomplete retrieval of identified research, reporting bias).	11
Conclusions	26	Provide a general interpretation of the results in the context of other evidence, and implications for future research.	11-12
**FUNDING**			
Funding	27	Describe sources of funding for the systematic review and other support (e.g., supply of data); role of funders for the systematic review.	14

From: Moher D, Liberati A, Tetzlaff J, Altman DG, The PRISMA Group (2009). Preferred Reporting Items for Systematic Reviews and Meta-Analyses: The PRISMA Statement. PLoS Med 6(6): e1000097. doi:10.1371/journal.pmed1000097

For more information, visit: **www.prisma-statement.org**.
